# Prognostic and functional role of subtype‐specific tumor–stroma interaction in breast cancer

**DOI:** 10.1002/1878-0261.12107

**Published:** 2017-08-22

**Authors:** Giuseppe Merlino, Patrizia Miodini, Maurizio Callari, Francesca D'Aiuto, Vera Cappelletti, Maria Grazia Daidone

**Affiliations:** ^1^ Biomarker Unit Department of Experimental Oncology and Molecular Medicine Fondazione IRCCS Istituto Nazionale dei Tumori Milano Italy

**Keywords:** breast cancer, gene expression profiling, microenvironment, prognosis

## Abstract

None of the clinically relevant gene expression signatures available for breast cancer were specifically developed to capture the influence of the microenvironment on tumor cells. Here, we attempted to build subtype‐specific signatures derived from an *in vitro* model reproducing tumor cell modifications after interaction with activated or normal stromal cells. Gene expression signatures derived from HER2+, luminal, and basal breast cancer cell lines (treated by normal fibroblasts or cancer‐associated fibroblasts conditioned media) were evaluated in clinical tumors by *in silico* analysis on published gene expression profiles (GEPs). Patients were classified as microenvironment‐positive (μENV+ve), that is, with tumors showing molecular profiles suggesting activation by the stroma, or microenvironment‐negative (μENV−ve) based on correlation of their tumors' GEP with the respective subtype‐specific signature. Patients with estrogen receptor alpha (ER)+/HER2−/μENV+ve tumors were characterized by 2.5‐fold higher risk of developing distant metastases (HR = 2.546; 95% CI: 1.751–3.701, *P* = 9.84E‐07), while μENV status did not affect, or only suggested the risk of distant metastases, in women with HER2+ (HR = 1.541; 95% CI: 0.788–3.012, *P* = 0.206) or ER‐/HER2− tumors (HR = 1.894; 95% CI: 0.938–3.824; *P* = 0.0747), respectively. In ER+/HER2− tumors, the μENV status remained significantly associated with metastatic progression (HR = 2.098; CI: 1.214–3.624; *P* = 0.00791) in multivariable analysis including size, age, and Genomic Grade Index. Validity of our *in vitro* model was also supported by *in vitro* biological endpoints such as cell growth (MTT assay) and migration/invasion (Transwell assay). *In vitro*‐derived gene signatures tracing the bidirectional interaction with cancer activated fibroblasts are subtype‐specific and add independent prognostic information to classical prognostic variables in women with ER+/HER2− tumors.

**Abbreviations**
αSMA: αsmooth muscle actin
BC: breast cancer
CAF: cancer‐associated fibroblast
CM: conditioned medium
DE: differentially expressed
DMFS: distant metastasis‐free survival
μENV: microenvironment gene expression signature
ER: estrogen receptor
FAP: fibroblast‐activation protein
FBM: fibroblast basal medium
FC: fold change
GGI: Genomic Grade Index
GSEA: gene set enrichment analysis
HR: hazard ratio
NAF: human fibroblast cell line
PgR: progesterone receptor

## 1 Introduction

Breast cancer (BC) is the most frequently diagnosed malignancy and the leading cause of cancer‐related death in women after lung cancer (Siegel *et al*., 2016). Clinical breast tumors are very heterogeneous with at least three subtypes with distinct biological features characterized by different clinical outcome (Parker *et al*., 2009). Approximately 90% of breast tumors are diagnosed at an early stage; nonetheless, still about 30% of women eventually relapse, depending on lymph nodal status and the tumor molecular features. This has prompted the introduction of adjuvant treatments which, as recommended by earlier guidelines (Harris *et al*., 2007), are guided by single biomarkers such as estrogen and progesterone receptors (ER and PgR) and HER2 or by gene panels, as more recently recommended (Harris *et al*., 2016), but still lead to treatment of a large fraction of women who would have never relapsed, with consequent morbidities, systemic toxicities, and increased costs for the health system. More recently, the results from the MINDACT trial addressed the overtreatment issue by identifying a large group of women for which 5‐year distant metastasis‐free survival was good even regardless of adjuvant chemotherapy administration (Cardoso *et al*., 2016), hopefully opening a new era when women might be spared unnecessary treatment based on the genomic profile of their tumors (Schmidt, 2016).

The behavior of tumor cells depends on their intrinsic features and on the interaction with the microenvironment composed by the basement membrane and the surrounding stroma, which is predominantly constituted by fibroblasts (Buchsbaum and Oh, 2016; Cunha, 1994; Donnarumma *et al*., 2017; Howlett and Bissell, 1993; Soysal *et al*., 2015), but also contains different populations of immune cells. Depending on their proximity to the tumor cells, fibroblasts undergo phenotypic and functional modifications becoming ‘activated myofibroblasts’ (CAFs). This creates a sustained fibrosis and wound healing response leading to a desmoplastic reaction which is in fact frequently observed in advanced breast carcinomas (Dvorak *et al*., 1981; Luo *et al*., 2015). Indeed, fibroblasts and their activation represent a considerable part of the host reaction in response to the local damage caused by the emerging cancer cells (Casbas‐Hernandez *et al*., 2011; Kalluri, 2016).

Contrary to their normal counterparts, CAFs express several mesenchymal markers, such as α‐smooth muscle actin (α‐SMA), fibroblast‐activation protein (FAP), and vimentin, have an increased rate of proliferation, produce distinct ECM proteins, and present an increased synthesis and release of several cytokines and growth factors (Gabbiani, 2003; Kalluri and Zeisberg, 2006). The reciprocal communication between cancer cells and fibroblasts is mainly mediated by the paracrine action of cytokines and growth factors: Among those, IL‐6 has already been identified as main actors in such an interaction. IL‐6 level, an important pro‐inflammatory cytokine mainly released by CAF, correlates with advanced tumor stage (Kozlowski *et al*., 2003), increased number of metastatic sites (Salgado *et al*., 2003), and poor prognosis (Bachelot *et al*., 2003; Salgado *et al*., 2003; Zhang and Adachi, 1999). This cytokine allows the normal fibroblasts to acquire the ‘activated’ phenotype (Giannoni *et al*., 2010).

Recently collected evidences provided an insight into the molecular mechanisms underlying interaction with the microenvironment, showing that fibroblasts affect many aspects of cancer cell behavior including proliferation, survival, angiogenesis, invasion, metastasis, and drug resistance (Wadlow *et al*., 2009), ultimately influencing the clinical outcome in breast cancer patients (Egeblad *et al*., 2005; Merlino *et al*., 2016). Interestingly, fibroblasts from different patients, from negative and positive lymph nodes (Riedel *et al*., 2016 and Santos *et al*., 2011), or from different anatomical sites (Wadlow *et al*., 2009) had a distinct effect on cancer cells.

These observations allowed us to hypothesize that searching in clinical tumors molecular alterations consequent to the interaction with the microenvironment might allow the identification of a signature recapitulating the microenvironment effect and informing on prognosis. We therefore investigated the context‐specific interactions of tumor cells with components of tumor microenvironment by *in vitro* models using breast cancer cell lines with distinct molecular subtypes. The *in vitro* model was used to generate three *subtype‐specific microenvironment gene expression signatures (*μ*ENV)* which were tested in different clinical settings by resorting to published transcriptomic datasets and demonstrated a prognostic value in the luminal context. To further support the validity of the model, we also report results on preclinical experiments investigating the biological effects of the tumor–microenvironment interaction.

## 2 Materials and methods

### 2.1 Experimental models

Human breast cancer cell lines (BCCLs) MCF7, SkBr3, MDA‐MB‐231, MDA‐MB‐361, MDA‐MB‐468, BT20, ZR75.1, T47D, and BT474 were purchased from American Type Culture Collection (ATCC) and cultured in Dulbecco's modified Eagle's medium (Lonza, Slough, UK) supplemented with 5% fetal bovine serum (Lonza). The human fibroblast cell line NHDF (NAF), derived from human normal derma (Lonza), and a cancer‐associated fibroblast (CAF) cell line, kindly provided by Bussolino, were cultured in fibroblast basal medium (FBM) supplemented with Fibroblast Growth Medium‐2 (FGM‐2) Bullet kit (Lonza), containing 2% FBS, 0.1% insulin, 0.1% GA‐1000, and 0.1% FGF. Cell lines were cultured at 37 °C in 95% humidified air in the presence of 5% CO_2_, and cell vitality (≥ 95%) was assessed by trypan blue exclusion assay before starting experiments. Authentication of cell lines by STR DNA profiling analysis was performed by the Genomic Core Facility at Fondazione IRCCS Istituto Nazionale Tumori (INT) before starting the experiments. For all ATCC cell lines, passage numbers of the original vials were available. Experiments were run thawing one vial from a cell bank prepared at the time of cell line purchase within two passages from the original one. All experiments were run within 5 to 10 passages from thawing of our cell bank vial. Our laboratory adopts a *Mycoplasma* contamination testing policy using an ELISA approach (MycoAlert mycoplasma detection kit; Lonza).

### 2.2 Conditioned medium collection

For collection of conditioned medium (CM), 550 000 fibroblasts (NAFs or CAFs) were plated in F25 flask, in DMEM F/12 5% FBS + FBM 2% FBS medium at a ratio 1 : 1 (hereafter referred to as MIX medium). After 24 h, the medium was replaced with 7 mL of serum‐free MIX medium and was collected at 72 h. CMs by NAFs and CAFs were clarified by centrifugation at 1400 × ***g*** for 3 min and used to treat for 72 h each BCCL plated in 24‐well plates (Corning) at a density of 150 000 cells.

### 2.3 Invasion and migration assays

Invasion and migration assays were performed in 24‐well plates using 6.5‐mm Transwell™ with 8.0‐μm Pore Polycarbonate Membrane Insert (Costar, Corning Life Science, NY, USA). See Doc. S1 for a detailed description of the assays.

### 2.4 Cell growth assay

BCCLs were seeded on the bottom of 96‐well plates at 7 × 10^3^ cells/well in 150 μL of serum medium and incubated at 37 °C in a humidified atmosphere of 95% air and 5% CO_2_. After 24 h, the medium was removed and the cells were washed with PBS to completely eliminate serum before treatment with CM. Cells were grown for 6 days, and cell growth was evaluated by MTT assay (see Doc. S1 for more details).

### 2.5 Cytokine quantification by ELISA

Determination of IL‐6 and IL‐8 concentration in the CM obtained from cocultures of BCCLs with NAF and CAF was performed by Quantikine^®^ High Sensitivity ELISA (R&D Systems, Abingdon, UK).

### 2.6 mRNA expression profiling

Total RNA was isolated from all BCCLs, treated for 24 h with CM derived from NAF/CAF, using Qiazol (Qiagen, Valencia, CA, USA). For microarray hybridization, 300 ng of total RNA was reverse‐transcribed, labeled with biotin, and amplified overnight (14 h) using the Illumina RNA TotalPrep Amplification kit (Ambion, Austin, TX, USA) according to the manufacturer's protocol. We collected primary data using the supplied scanner software, and the microarray raw data were obtained using illumina beadstudio 3.1.3.0 software and processed using the *lumi* Bioconductor package (Riedel *et al*., 2016). After quality control, the robust spline normalization was applied on log2‐transformed data, and probes with a detection *P* < 0.01 in at least one sample were selected. For each gene, the probe with the highest detection rate was chosen, or with equal detection rates, the one with the highest interquartile range. Raw and processed data were deposited to the Gene Expression Omnibus data repository (Barrett *et al*., 2011) with ID GSE70884.

### 2.7 Analysis of transcriptomic data

On data obtained from BCCLs, class comparison analysis was performed using the *limma* Bioconductor package (r version 2.15.2; Du *et al*., 2008), and a two‐tailed *P*‐value < 0.0001 was considered as statistically significant. A biological cutoff was set for identification of differentially expressed (DE) genes by considering only FC < 0.67 or FC > 1.5. At a *P*‐value = 0.0001, 2.2 genes are expected to be deemed as DE by chance.

Gene set enrichment analysis was performed using gsea (Subramanian *et al*., 2005). A custom collection including canonical pathway (Kegg, Biocarta, and Reactome) and gene ontology terms was tested on the gene list ranked according to *t‐*statistic values obtained from class comparison analysis. Terms with FDR < 0.05 were considered significant.

Nine publicly available datasets downloaded from the Gene Expression Omnibus website (Barrett *et al*., 2011) or ArrayExpress website (Rustici *et al*., 2013) were collected and categorized as follows: PROGNOSTIC collection includes a total of 826 gene expression profiles of primary‐node‐negative breast cancer from patients not receiving systemic treatment, and TAM and CHEMO collections include a total of 685 and 537 gene expression profiles of primary tumors from patients receiving adjuvant endocrine therapy or chemotherapy, respectively (Callari *et al*., 2016).

All collected gene expression data were derived from the Affymetrix GeneChip Human Genome U133A or U133 Plus 2.0 platforms. Raw signals were imported using *affy* Bioconductor package (Gautier *et al*., 2004) and processed as previously described (Callari *et al*., 2016).

Patients were subdivided into three subtypes based on *ESR1* and *ERBB2* expression levels in a way similar to that described in Bianchini *et al*. (2010): *ESR1*−*/ERBB2*− (roughly corresponding to basal‐like subtype), *ESR1+/−/ERBB2+* (roughly corresponding to HER2+ enriched subtype), and *ESR1+/ERBB2*− (roughly corresponding to luminal A and B subtypes). The NM_000125 (probeset 205225_at) and NM_001005862 (probeset 216836_at) reference sequences were considered as reporters for *ESR1* and *ERBB2*, respectively, and the threshold values were defined based on the strong bimodal distribution observed (Callari *et al*., 2014).

### 2.8 Statistical analysis

A major endpoint of this study was to assess the association between microenvironment signatures specific for ER+/HER2−, HER2+, and ER−/HER2− BCCLs and disease‐free survival in the series of 2048 patients belonging to the PROGNOSTIC, TAM, and CHEMO datasets (Callari *et al*., 2016) and classified in the three different subtypes. Each signature was dichotomized as described in Results and investigated in the corresponding breast cancer subtype; Kaplan–Meier curves were plotted using the same cutoff, and survival differences were estimated by using the log‐rank test. Univariable and multivariable Cox proportional hazards regressions, as implemented in the *survival* package (r version 2.15.2), were used to correlate subtype‐specific μENV signature with clinical outcome in the patient collection. Proportional hazard assumptions were evaluated using a goodness‐of‐fit testing procedure based on Schoenfeld residuals (Schoenfeld, 1982).

Data showing the biological effects on BCCLs of CAF/NAF CM treatment are reported as mean values ± standard deviation from at least three independent experiments, and statistical analysis was performed by two‐tailed Student's *t*‐test. *P*‐values < 0.05 were considered statistically significant.

## 3 Results

The role of the microenvironment in modulating tumor progression was studied in BCCLs belonging to different subtypes after paracrine interaction with NAFs and CAFs.

**Figure 1:**
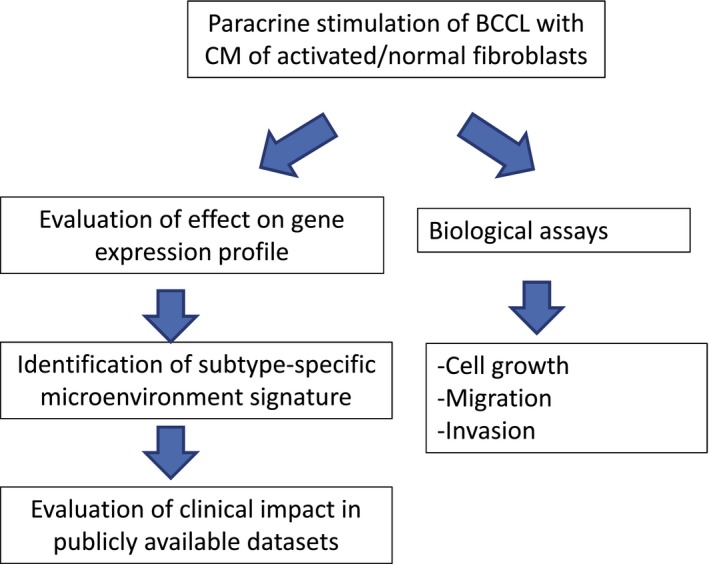
Workflow of the analysis.

Genes specifically modulated by CAFs were used to derive three subtype‐specific gene signatures, which were tested in public gene expression datasets of primary tumors for their association with clinical outcome. In parallel, microenvironmental effects on cell growth, migration, and invasion were evaluated in a subset of cell lines by functional assays. An outline of the different phases of the study is reported in Fig. 1. All cell line experiment results were derived from biological independent triplicates.

### 3.1 Transcriptome alterations of BCCLs after interaction with fibroblasts

The interaction of the ‘normal’ (represented by NAF cells) and activated (represented by CAF) stromal compartments with the tumor was modeled by treating epithelial cell lines with culture media conditioned by fibroblasts. The paracrine effects exerted by fibroblasts on the epithelial cells were evaluated at transcriptome level by running gene expression profiles in nine cell lines, three basal‐like (including the two basal‐A‐like cell lines MDA‐MB‐468 and BT20 and the basal B‐like/claudin‐low cell line MDA‐MB‐231), three HER2+ (SkBr3+; BT474; and MDA‐MB‐361), and three luminal cell lines (T47D; MCF7; and ZR75.1), after treatment with either CAF or NAF CM for 72 h. Molecular subtype of cell lines was attributed according to Neve *et al*. (2006) and Heiser *et al*. (2012).

**Figure 2:**
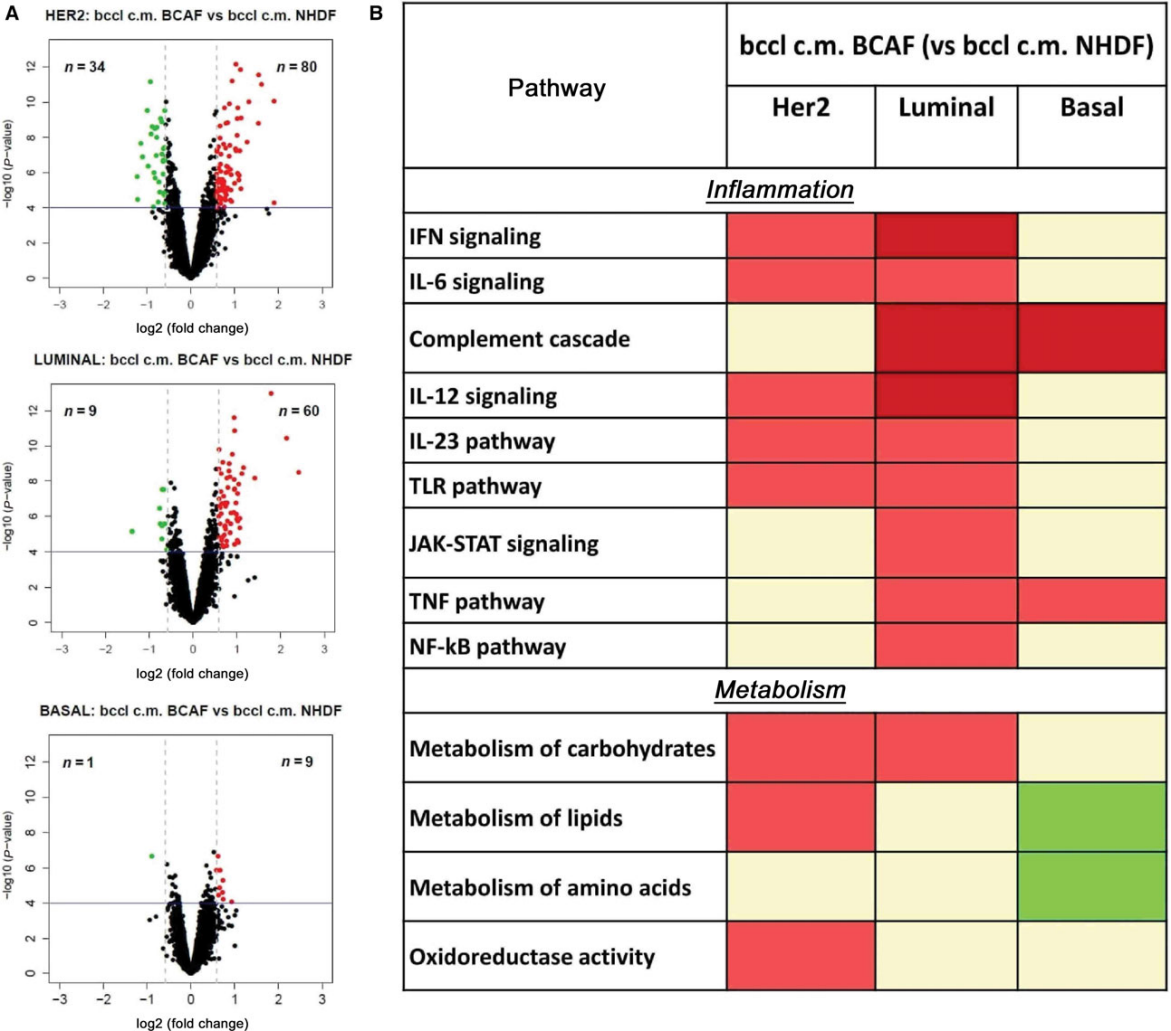
Effects of microenvironment upon gene expression profile of breast cancer cell lines (BCCLs). (A) Volcano plots for differentially expressed genes between BCCLs treated with conditioned media (CMs) from activated fibroblasts and BCCLs treated with conditioned media from normal fibroblasts. The green and red boxes respectively indicate down‐ and upregulated genes using as significance thresholds *P* < 0.0001 and FC < 0.67 or FC > 1.5. (B) Pathways significantly enriched after treatment with BCAF CM (respect to NHDF CM) in each subtype. Significantly enriched gene sets were identified by GSEA and grouped based on biological function. The lowest FDR is reported for each group using a color code. Red boxes indicate positively enriched pathways (dark and light red indicate FDR < 0.001 and 0.001 ≤ FDR < 0.005, respectively); green boxes indicate negatively enriched pathways (dark and light green indicate FDR < 0.001 and 0.001 ≤ FDR < 0.005, respectively). All gene expression results refer to biological independent triplicates for each test condition.

By class comparison approaches, CAF vs NAF CM‐induced gene modulations were investigated at subtype level. The number of differentially expressed (DE) genes, identified at the same significance thresholds (*P*‐value < 1E‐04 and FC < 0.67 or FC > 1.5), was higher in luminal and HER2+ subtypes (69 and 114 genes, respectively) compared to basal‐like cell lines (10 genes). Furthermore, in each cell group the majority of DE genes were defined as upregulated rather than downregulated (Fig. 2A). The complete gene list is provided in Doc. S1 and includes both DE genes derived from class comparison of CAF vs NAF (used for deriving our signature) and DE genes in CAF vs serum‐free control medium and NAF vs serum‐free control medium (included for completeness).

As a next step, we evaluated common and subtype‐specific altered pathways by performing gene set enrichment analysis (GSEA) on the genes identified by class comparison. Genes were ranked with respect to *t*‐statistics obtained from class comparison analysis. The specific enrichment for functionally related genes was tested across a collection of 1843 curated gene sets including the canonical pathways (Kegg, Biocarta, and Reactome, included in C2 collection) and gene ontology terms (C5 collection). By this analysis, pathways positively or negatively associated with CAF CM (vs NAF CM) treatment were identified. To attain a biological interpretation of these findings, gene sets used for GSEA were subgrouped into different categories linked with specific pathways or with biological functions (Fig. 2B).

Several pathways, such as interferon signaling, IL‐6, IL‐12 and IL‐23 signaling, Toll‐like receptor pathway, pathways related to inflammation, and metabolism of carbohydrates, were positively enriched in both HER2+ and luminal subtypes. Among pathways positively enriched in luminal and basal subtypes, we observed the complement cascade and tumor necrosis factor pathways. No common pathway was observed between HER2+ and basal subtypes.

In HER2+ cell lines, we identified a positive enrichment for lipid metabolism and oxidoreductase activity, whereas in luminal subtype, JAK–STAT signaling and NF‐κB pathways were positively enriched. Only in the basal subtype, the stimulation with CAF CM (vs NAF CM) caused a negative enrichment of lipid and amino acid metabolism.

### 3.2 Identification of subtype‐specific microenvironment signatures (μENV)

Differentially expressed genes identified by class comparison analysis between luminal HER2+ and basal BCCLs treated with CM from CAF or from NAF were used for building three different microenvironment signatures representative of stromal effect on breast cancer cells belonging to the three different subtypes (μENV_lum_, μENV_HER2_, and μENV_basal_). The reference levels for each subtype‐specific signature were obtained by calculating the mean expression of DE genes in BCCLs representative of the specific subtype, treated with CM obtained from CAFs. These values were then used to classify individual clinical samples with respect to the degree of microenvironment‐related gene stimulation (hereafter referred to as μENV status).The ‘microenvironment status’ of clinical samples was defined by Spearman's correlation between defined subtype‐specific signatures and the gene expression levels in patients with the corresponding tumor subtype. Tumors with correlation value higher than 0 were classified as ‘microenvironment‐positive’ (μENV+).

To remove the dependence on magnitude of gene expressions between different datasets used (experimental data and case series), each collection was mean‐centered by subtracting from expression value of each gene its average signal of all samples on the platform.

Before assessing the clinical relevance of the identified signatures, their overlap with previously described microenvironment‐related signatures was evaluated. To such a purpose, we considered five signatures among those described in Giussani *et al*. (2015), namely the signatures by Chang *et al*. (2004), by West *et al*. (2005), by Helleman *et al*. (2008), by Farmer *et al*. (2009), and by Bergamaschi *et al*. (2008). The observed lack of overlap between our microenvironment‐related signatures and published data proved their novelty (Table S1).

### 3.3 Validation of μENV signatures by association with prognosis in publicly available datasets

The relevance of each μENV signature was evaluated in publicly available datasets with known clinical outcome. The pure prognostic relevance was explored exploiting the so‐called PROGNOSTIC dataset. Percentages of patients defined as μENV‐positive were different between the subtypes (43% among patients with ER+/HER2− tumors, 64% among patients with HER2+ tumors, and 79% among patients with ER−/HER2− tumors, *P *< 0.00001).

**Figure 3:**
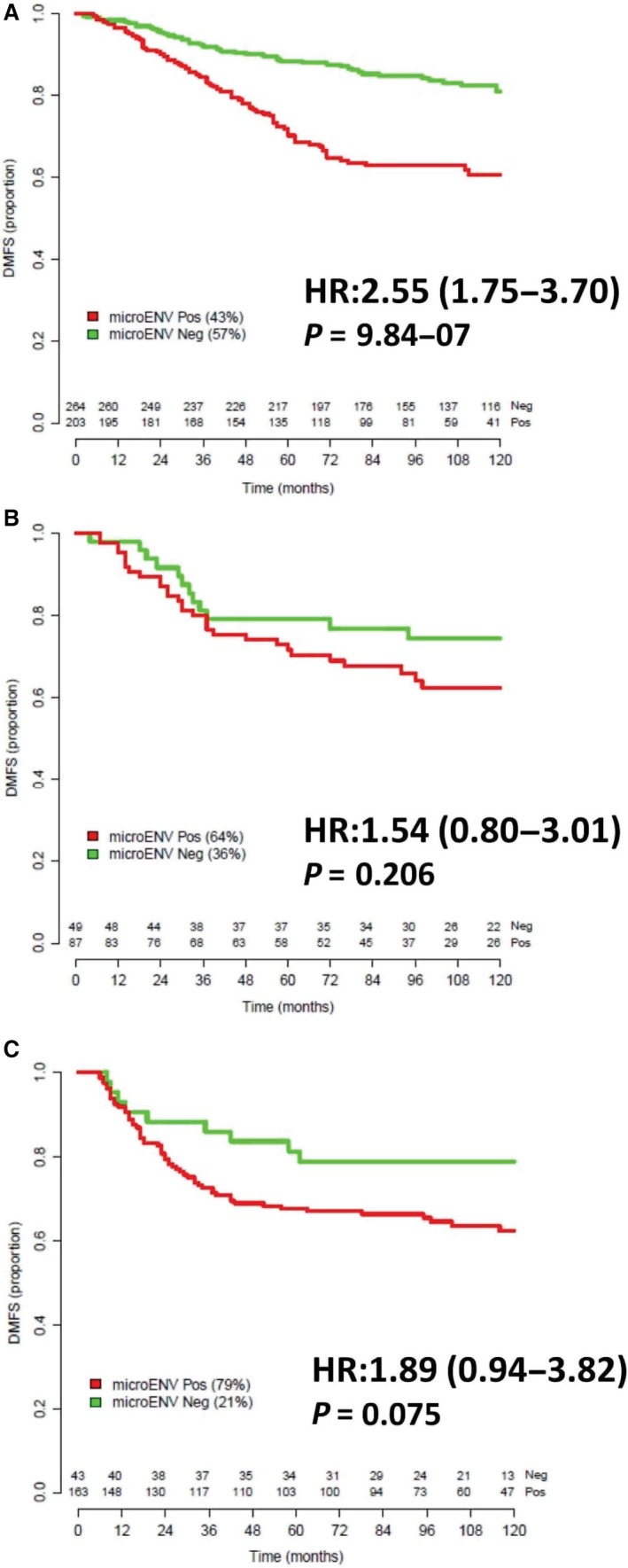
Kaplan–Meier curves comparing distant metastasis‐free survival (DMFS) according to μENV status defined using subtype‐specific μENV signatures. (A) patients with ERα+/ERBB2‐, (B) patients with ERBB2+ tumors, and (C) patients with ERα‐/ERBB2‐ tumors. The number of patients in each group at each time is reported. For each comparison hazard ratio (HR), 95% CI and *P‐*value are reported.

For each subgroup of patients (Table S2), Kaplan–Meier curves were plotted (Fig. 3). Women bearing ER+/HER2− tumors, defined as μENV+ve, showed a reduced DMFS compared to those with μENV−ve tumors (*P *= 9.84E‐07) and a more than 2.5‐fold higher 10‐year recurrence risk. A similar finding, although of marginal statistical significance (*P *= 0.0747), was obtained for ER−/HER2− tumors, where the 10‐year recurrence risk was still almost twofold higher in patients with μENV+ve tumors, although the wide 95% confidence interval does not significantly support the relevance of this finding. Conversely, in women with tumors classified as HER2+, DMFS curves were not affected by the μENV signature.

The subtype dependence of the generated μENV signatures on DMFS was tested by univariable and survival curves analyses employing each of the signatures out of their subtype context. Data are reported in Fig. S1. For all the explored combinations, DMFS proved to be unaffected by μENV signatures unrelated to the specific subtype.

As shown in Table 1, the luminal μENV signature maintained its independent prognostic significance on DMFS in women with ER+/HER2− tumors even in the presence of well‐known prognostic factors such as size, age (evaluated as dichotomous variables), and Genomic Grade Index, GGI (Sotiriou *et al*., 2006) (HR = 2.098; CI: 1.214–3.624; *P* = 0.00791). As GGI did not satisfy the proportional hazard assumptions (Table S3), in the Cox regression the GGI‐by‐time interaction was included.

**Table 1:** Multivariable Cox regression analysis in lymph‐node‐negative untreated patients with ESR1+/ERBB2‐ tumors (297 patients, 53 unfavorable events)

Variable	HR (95% CI)	*P*‐value
μENV (+ve, −ve)	2.098 (1.214–3.624)	0.00791
Age (> 50, ≤ 50)	1.334 (0.752–2.367)	0.325
Size (> 2 cm, ≤ 2cm)	2.093 (1.194–3.669)	0.00990
GGI (> 0, ≤ 0)	6.214 (3.076–12.55)	3.54E‐07
GGI: gtime	0.112 (0.027–0.466)	0.00262

Finally, we tested whether the women with μENV+ve tumors were not characterized by a worse prognosis due to an overlap with luminal B tumors. Using the PAM50 signature (Parker *et al*., 2009), we classified patients of our prognostic dataset into luminal A and luminal B. Logistic regression of the μENV signature using the luminal A/B classification as dependent variable did reveal a statistically significative association (OR = 0.04; *P* < 0.001), but with lower μENV metagene levels in the luminal B group than in the luminal A group. This result does not support the possibility that women with μENV+ tumors have a worse prognosis due to an enrichment in luminal B subtype.

As luminal B tumors are distinguished from luminal A tumors mainly due to high proliferation, it also suggests that proliferation is not the main driver for the prognostic relevance of the μENV signature.

We next evaluated the performance of our μENV signatures for luminal tumors with respect to treatment, using the CHEMO and TAM collections as described in Materials and methods. In 189 women belonging to the CHEMO dataset, the 10‐year HR for developing distant metastases was unrelated to the μENV signature (HR = 1.06; 95% CI 0.58–1.95, *P *= 0.853). Similar results were observed in 523 women receiving adjuvant treatment with tamoxifen (HR = 1.40; 95% CI 0.93–2.10, *P *= 0.107).

### 3.4 *In vitro* assay for investigating the interaction of breast cancer cell lines and fibroblasts

Microenvironment–cancer cell interactions were studied from a functional point of view by focusing on one cell line as representative of each subtype: SkBr3 cells for HER2+ subtype, T47D for luminal subtype, and MDA‐MB‐468 for basal subtype.

All the collected CMs were characterized for their content of two selected cytokines (IL‐8 and IL‐6) using an ELISA kit. Figure S2 reports the results summarized in a heat map to simplify comparisons. When grown alone, SkBr3 and T47D and the normal fibroblasts, NHDF, did not release in the CM neither IL‐8 nor IL‐6, whereas on the opposite, the basal A MDA‐MB‐468 cells and the CAF cells released high levels of both interleukins. The direct interaction of NAF with the two T47D or SkBr3 cell lines caused an increased release of interleukins, especially for IL‐6. The release was even higher in the case of interaction with CAF. The interaction of MDA‐MB‐468 with normal or activated fibroblast did not modify the release of IL‐6 and IL‐8 already released at high levels by all of these cells when separately cultured. Therefore, whereas the reported results do not allow identification of which cell line is responsible for the increased interleukin release, they clearly show that the tumor microenvironment is definitely modified with respect to its cytokine content upon an interaction between tumor and stromal cells (especially if activated; Fig. S2).

**Figure 4:**
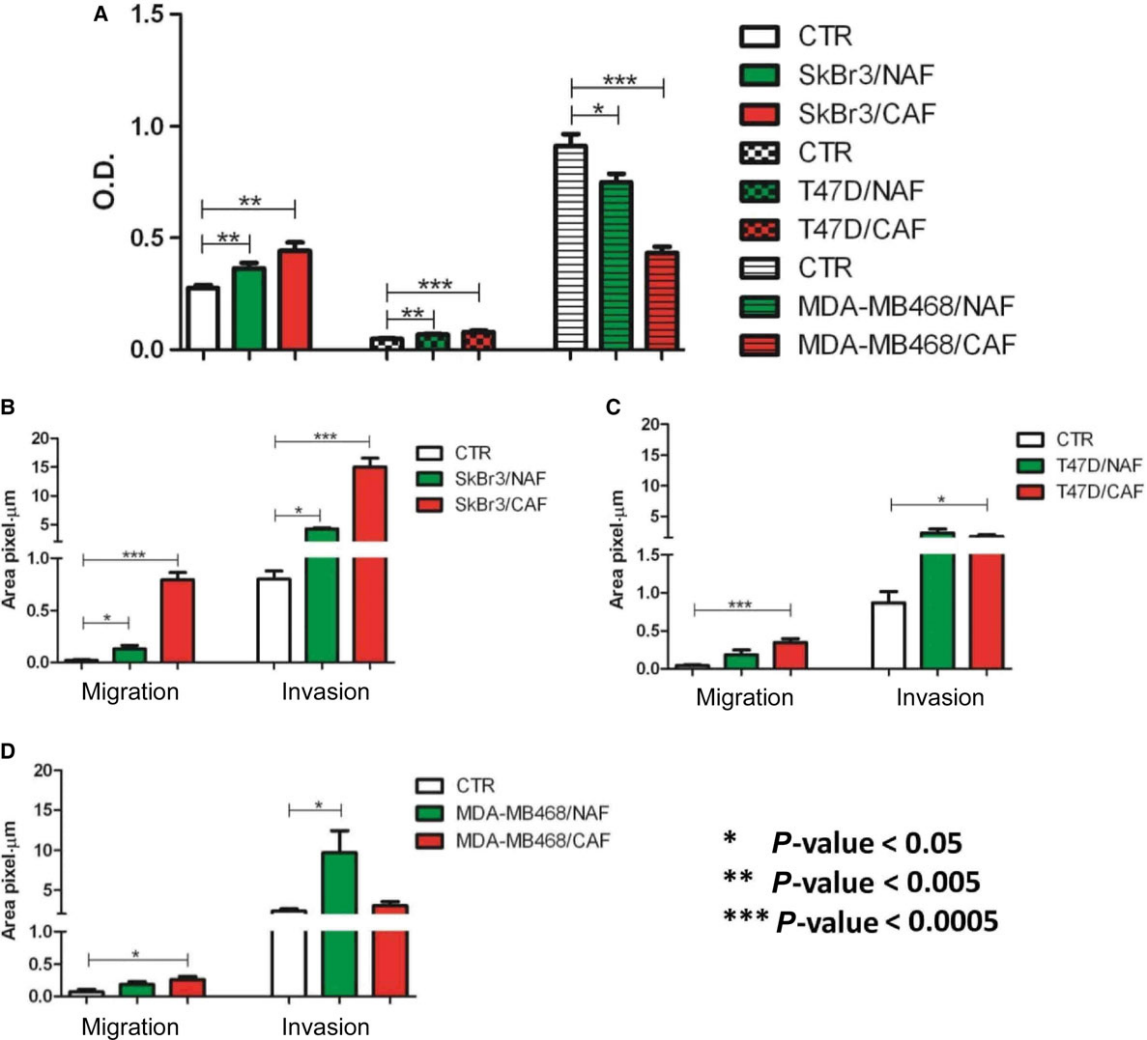
*In vitro* assays on cell growth, migration, and invasion. (A) Growth of SkBr3, T47D, and MDA‐MB‐468 cells upon stimulation with conditioned medium (CM) from NAFs and CAFs. Bars represent mean optical density units (OD) as obtained from three independent experiments by MTT assays ±SD. (B–D) Migration and invasion assays of SkBr3, T47D, and MDA‐MB‐468, respectively, cocultured with NAFs and CAFs. Bars represent the mean fraction of sulforhodamine‐stained cells per area unit as detected by microscope examination from three independent experiments ±SD. Statistical significance of differences was evaluated by Student's *t*‐test. *P < 0.05; **P < 0.005, ***P < 0.0005.

To investigate with more detail the effect of NAF and CAF on BCCL, we set up biological assays for assessing growth in cells treated with CM and Transwell experiments for migration and invasion (Fig. 4).

Culture media conditioned by NAF or CAF significantly (*P *< 0.005) stimulated SkBr3 and T47D growth, evaluated at 7 days by MTT assay. Different from the HER2+ and luminal cell lines, the CM of both NAF and CAF did not increase, but reduced (*P *< 0.005) the growth of MDA‐MB‐468 (Fig. 4A).

The migration and invasion capacity of BCCLs, upon the interaction with fibroblasts, was evaluated in Transwell heterotypic cocultures, seeding the fibroblasts on the bottom of the well and the tumor cells inside the Transwell™.

Migration and invasion of SkBr3 were significantly stimulated by both NAF and CAF (Fig. 4B). For both the evaluated endpoints, the effect exerted by CAFs was stronger compared to NAFs, in keeping with the cytokine release results (Fig. S2).Treatment with NAF or CAF CM also increased the migration (*P *< 0.0005) and invasion (*P* < 0.05) of T47D cells (Fig. 4C), whereas the basal A cell line MDA‐MB‐468 positively reacted to the interaction with CAFs, but not with NAFs when migration was evaluated (Fig. 4D). Conversely, in the case of invasion there was a significant (*P *< 0.05) stimulation by NAFs and not by CAFs.

## 4 Discussion

Tumors are seen as heterogeneous diseases characterized by the acquirement of somatic mutations and undergoing clonal evolution (Greaves and Maley, 2012). Consequently, tumors present with deregulations in numerous pathways and with a malignant phenotype characterized by distinct gene expression profiles. However, we know that in such a process, the microenvironment is not a simple bystander (Hanahan and Coussens, 2012) as it is able to modify cell shape, tissue organization, and obviously also gene expression and cell behavior. As in the bidirectional communication between cells and their microenvironment, fibroblasts are among the main actors, although not the only ones, we hypothesized that a gene expression signature recapitulating such an interaction could inform on tumor progression and predict metastatic spread. We know however that stromal signatures derived from clinical tumors contain genes associated with different subpopulations and not only fibroblasts, including endothelial, inflammatory, and immune cells as recently described by Winslow *et al*. (2015).

Being aware of the well‐known association between prognosis and molecular subtype and of the distinct signaling pattern of each molecular subtype, we decided to develop subtype‐specific signatures rather than a unique signature for all tumor types. We therefore built *in vitro* models, from which three distinct cancer cell gene expression signatures specific for luminal, HER2‐positive, and basal tumors were derived and subsequently challenged as prognostic biomarkers in clinical tumors. Whereas pure stromal signatures have been described in the literature (Beck *et al*., 2008 and Finak *et al*., 2008), to the best of our knowledge, no signatures reflecting the state of tumor cells upon interaction with an activating microenvironment have been reported.

The subtype specificity of our signatures represents an innovation compared to literature data and highlights the fact that interaction with the microenvironment involves activation of distinct pathways based on biological features of tumor cells. In particular, tumors classified as ER−/HER− do not seem to sense the influence of an activated microenvironment and might be in certain way considered as constitutively activated. In fact, almost 80% of tumors belonging to this subtype were classified as μENV+ve according to the specific signature. On the contrary, HER2+ tumor cells are modified upon interaction with activated fibroblasts, but those changes do not seem to affect prognosis in women with HER2+ tumors, suggesting that the tumor‐intrinsic features play a major role compared to the microenvironment. ER+/HER2− cancer cells, instead, sense the microenvironment activating inflammatory and metabolic pathways, and the resulting transcriptional changes, when detected in clinical tumors, are associated with a grim prognosis for women with ESRI+/ERBB2− tumors. ER+/HER2− tumors appear to be therefore not only estrogen‐responsive but also ‘microenvironment‐sensitive’. Nonetheless, in women with such tumors in the presence of either anti‐estrogens or standard chemotherapy treatment, differences in risk of metastasis as a function of microenvironment signature were not observed. We might speculate that the μENV signature has a prognostic relevance rather than predictive relevance; however, in the absence of randomized trial with a treatment and a control arm, no definitive conclusion can be drawn on its predictive role.

The specificity of our μENV signatures was also supported by their complete lack of prognostic relevance out of their subtype context. A strength point in our approach comes from the successful way of integrating previous knowledge from the literature (van't Veer *et al*., 2002) on the biological and clinical differences between breast tumors in the context of new prognostic studies. This goes in the direction of searching a progressive refinement of prognostic tools by embedding previous knowledge to facilitate clinically relevant achievements. In early‐stage breast cancer, therapeutic planning considers clinicopathological variables as risk predictors, but is guided by subtype for the optimal treatment choice. Despite optimal matching of patient groups with treatments based on their tumor subtype, still a relevant percent of patients receive unnecessary treatments due to prognostic heterogeneity and could instead be spared the toxicity (Callari *et al*., 2016; Cardoso *et al*., 2016; Harris *et al*., 2016; Sparano *et al*., 2015). This problem, which is at the heart of the recently published results of large trials (MINDACT), has so far been addressed using prognostic signatures, however without specifically taking into account the important contribution of the stroma.

Nonetheless, the importance of the microenvironmental contribution to refine tumor‐derived prognostic signatures and classic clinic–pathologic prognostic factors has already been reported in the past. For instance in the study by Finak *et al*. (2008), a stroma‐derived prognostic predictor (SDPP) obtained from laser‐capture‐microdissected breast tumors was shown to stratify patients also when considering nonmicrodissected tumors independently of ER and HER2 status. Though, the SDPP by Finak and colleagues does capture a different dimension of the stromal prognostic space compared to the μENV signature, as SDPP contains genes associated with proficient immune, neo‐angiogenic, and hypoxic responses.

The choice of only two representative cells lines for NAFs and CAFs might represent a limitation in our study. We would however like to point out that our NAF and CAF representatives were chosen due their biological reliability. In fact, our CAF cells overexpress the same genes and the same gene categories as do activated fibroblasts derived from other tumor types such as prostate and lung (Gandellini *et al*., 2015), suggesting a common path in stromal activation in solid tumors. Furthermore, we have previously observed that the tumor subtype does not impact the pattern of paracrine‐mediated activation of fibroblasts (Merlino *et al*., 2016), thus suggesting that selection of fibroblasts isolated from clinical tumors with different molecular phenotypes is not strictly necessary to obtain a reliable gene signature.

The clinical relevance of our signatures, which is demonstrated in public datasets, was in a sense both predicted and supported by our *in vitro* data. In fact, in keeping with the weak clinical relevance of the μENV signature in ER−/HER2− tumors, we did not observe neither stimulation of cell growth, migration, and invasion nor modification in the cytokine milieu of MDA‐MB‐468 cells upon interaction with fibroblasts. Functional modifications have been instead observed for luminal and for HER2‐+ve cells. Noteworthy, SkBr3 cells responded even better than T47D cells to paracrine stimulation exerted by fibroblasts, in terms of growth, migration, and invasion, despite the fact that the HER2‐specific μENV signature did not discriminate distant metastasis risk. A possible interpretation for this finding relates to a major role of other microenvironmental components in this tumor type, such as immune cells (Rody *et al*., 2009). The same observation could be applied also to ER−/HER2−, a subtype where the role of the immune response is well known (Rody *et al*., 2009; Schmidt *et al*., 2008; Teschendorff *et al*., 2007) and has also been recently reconfirmed by our group (Callari *et al*., 2016). Conversely, the importance of the stromal compartment in the luminal subtype has been reported also by other groups (Dennison *et al*., 2016).

The partial success in developing a distant metastasis risk predictor in ER−/HER2− tumors might also represent a limitation of this study. In the case of HER2+ tumors, the poor performance of the μENV signature might be related to intrinsic properties of HER2+ tumors as well as to the fact that within the HER2+ group no distinction is performed between ER+ and ER− cases. This applies both to the clinical tumors and to the cell lines chosen as representatives.

Finally, as in this study only paracrine‐mediated effects have been considered, we cannot exclude that in the case of HER‐2‐enriched or of basal tumors, a direct contact between heterotypic cells is necessary to elicit stimulation by the stroma.

The novelty of our approach lies instead in having shown that the bidirectional interaction with fibroblasts is subtype‐specific and that in the ER+/HER2− subtype the tracking of such an interaction has prognostic relevance. Future, more clinically addressed studies should however focus on combining such interaction‐focused signature with pure tumor‐focused signatures.

## Author contributions

GM and VC conceived and designed the project; GM and PM performed the experiments; MC and FD analyzed the data; GM, VC, and MGD interpreted the data; and GM, VC, and MGD wrote the manuscript.

## Data accessibility

## Supporting information


**Fig. S1.** Kaplan–Meier curves comparing distant metastasis‐free survival (DMFS) according to μENV status defined using non‐subtype‐specific μENV signatures.

**Fig. S2.** Heat map summarizing IL‐8 and IL‐6 levels in conditioned media (CMs) from monotypic and heterotypic cell cultures.

**Table S1.** Overlap between the μENV signatures and published microenvironment‐related signatures.
**Table S2.** Patient numbers and number of unfavorable events in the publicly available gene expression collections.
**Table S3**. Results of test based on Schoenfeld residuals for checking proportional hazards in multivariable Cox analysis for lymph‐node‐negative untreated patients with ESR1+/ERBB2‐ tumors.

**Doc. S1.** Supplementary methods.
