# Supplementary material for: Prognostic and functional role of subtype‐specific tumor–stroma interaction in breast cancer
Source: Mol Oncol. 2017 Aug 22;11(10):1399–412. doi: 10.1002/1878-0261.12107 (PMC5623822; doi:10.1002/1878-0261.12107)
Supplement: Supplementary file 1 — Fig. S1. Kaplan–Meier curves comparing distant metastasis‐free survival (DMFS) according to μENV status defined using non‐subtype‐specific μENV signatures. [file MOL2-11-1399-s001.pdf]

Supplementary Figure 1

Signature derived from **Luminal** cells

Signature derived from **Her2** cells

Signature derived from **Basal** cells

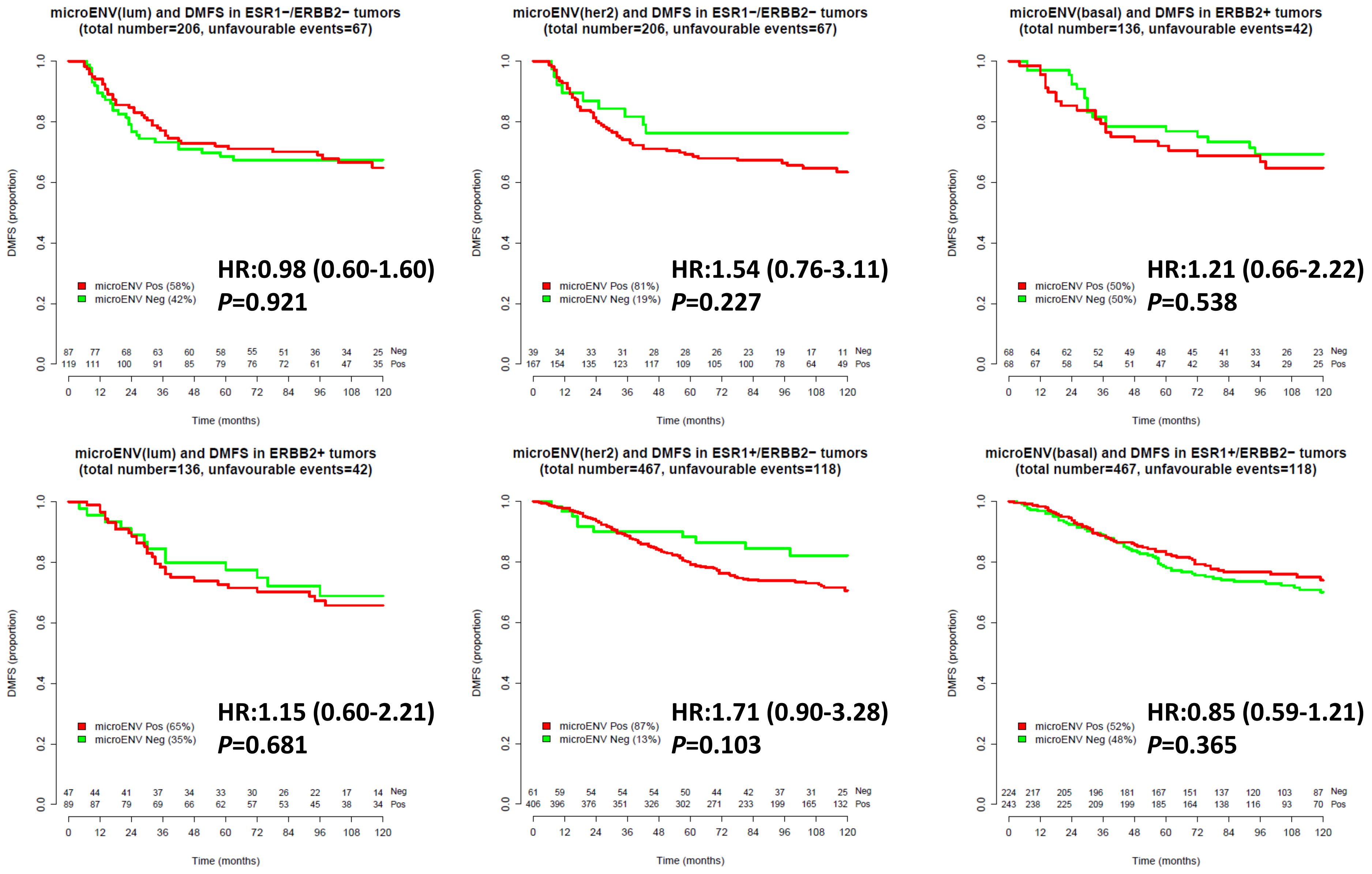

Supplementary Figure 2

	IL-8 in CM			IL-6 in CM		
	single	*NAF*	*B-CAF*	*single*	*NAF*	*B-CAF*
SkBr3	0.76	6.66	14.84	0.55	15.05	9.64
T47D	0.02	1.09	43.39	0.44	14.9	9.34
MDA-MB-468	62.58	64.79	68.93	11.13	12.26	11.23
NHDF	0.86	nd	nd	0.9	nd	nd
B-CAF	15.32	nd	nd	9.55	nd	nd
