# Supplementary material for: Prognostic and functional role of subtype‐specific tumor–stroma interaction in breast cancer
Source: Mol Oncol. 2017 Aug 22;11(10):1399–412. doi: 10.1002/1878-0261.12107 (PMC5623822; doi:10.1002/1878-0261.12107)
Supplement: Supplementary file 3 — Table S1. Overlap between the μENV signatures and published microenvironment‐related signatures. Table S2. Patient numbers and number of unfavorable events in the publicly available gene expression collections. Table S3. Results of test based on Schoenfeld residuals for checking proportional hazards in multivariable Cox analysis for lymph‐node‐negative untreated patients with ESR1+/ERBB2‐ tumors. [file MOL2-11-1399-s003.docx]

**Table S1.** Overlap between the µENV signatures and published microenvironment-related signatures

| **literature Gene signature** | **number of genes** | **% gene overlap with µENV signatures** | | |
| --- | --- | --- | --- | --- |
|  |  | **µENV basal** | **Her2** | **Lum** |
| Bergamaschi et al. J Pathol. 2008 | 58 | 0 | 0 | 0 |
| Chang et al. PLoS Biol 2004 | 108 | 0 | 0 | 0 |
| Farmer et al. Nat Med 2009 | 50 | 0 | 0 | 0 |
| Helleman et al CCR 2008 | 69 | 0 | 0 | 0 |
| West et al. PLoS Biol 2005 | 656 | 0 | 1.37 | 0.76 |

**Table S2.** Patient numbers and number of unfavorable events in the publicly available gene expression collections.

| **Gene expression collection** | **No. of patients** | **No. of unfavourable events** |
| --- | --- | --- |
| PROGNOSTIC |  |  |
| *ESR1*+/*ERBB2*- | 467 | 118 |
| *ERBB2*+ | 136 | 42 |
| *ESR1*-/*ERBB2*- | 206 | 67 |
| TAM | 523 | 97 |
| CHEMO | 189 | 42 |

**Table S3**. Results of test based on Schoenfeld residuals for checking proportional hazards in multivariable Cox analysis for lymph-node negative untreated patients with ESR1+/ERBB2- tumors

| **Variable** | **Rho** | **Chi-Square** | ***P*** |
| --- | --- | --- | --- |
| µENV | 0.082 | 0.414 | 0.520 |
| GGI | -0.346 | 7.207 | 0.007 |
| Age | 0.061 | 0.232 | 0.630 |
| Size | -0.212 | 2.656 | 0.103 |
