# Supplementary material for: Prognostic and functional role of subtype‐specific tumor–stroma interaction in breast cancer
Source: Mol Oncol. 2017 Aug 22;11(10):1399–412. doi: 10.1002/1878-0261.12107 (PMC5623822; doi:10.1002/1878-0261.12107)
Supplement: Supplementary file 4 — Doc. S1. Supplementary methods. [file MOL2-11-1399-s004.docx]

**In-vitro interaction between breast cancer cell lines and stroma: clinical validity of a microenvironment (µENV) subtype specific signature**

**Giuseppe Merlino, Patrizia Miodini, Maurizio Callari, Francesca D’Aiuto, Vera Cappelletti* and Maria Grazia Daidone**

Supplementary files

1. Supplementary Methods:

The file contains description of methodological details:

**Invasion and migration assays**

For the invasion assay, the membranes were coated with 60 μL of Matrigel™ (200 μg/mL) and air-dried at 4°C. The filters were re-hydrated with 200 μL of serum-free media at room temperature for 60 min before use. One hundred μL of medium containing 60,000 BCCLs were added on the top of Trans-chambers, seeded 24 hrs in advance with 90,000 fibroblasts in the lower chambers. The 24-well plates were incubated in a 5% CO_2_ humidified incubator at 37°C for 72 hrs. The cells on the upper surface were gently removed with a cotton swab and the filters were fixed with ethanol 100% for 30 minutes at -20°C and then washed with distilled water.

The migrated or invading cells were stained with sulforhodamine (SRB) for 30 minutes at room temperature, then SRB was removed and each well was washed with 1% acetic acid and dried overnight. The cells on the lower surface of the filters were detected by microscope examination; each test group was assayed in triplicate.

Migration assays were run in the same way, but omitting the Matrigel™ coating.

The migrating or invading cells were quantified using ImageJ™ software.

**MTT assay:**

Ten µL of MTT solution (5 mg/mL) (Sigma-Aldrich, St. Luois, MO, USA) were added to each well, and the cells were incubated for 4 hrs at 37° C. Formazan crystals were then dissolved in 150 μL DMSO (Sigma-Aldrich). The optical density (OD) was measured with a microplate reader (BioRad, Hercules, CA, USA) at 595 nm.

**Microarray hybridization**

**Isolated total RNA was subjected to a** clean-up treatment with RNAeasy kit following the manufacture’s recommendations (Qiagen, Valencia, CA) and with RNase-free DNase to remove contaminating genomic DNA, RNA integrity and purity was assessed by Bioanalyzer (Agilent). RNA concentration was spectrophotometrically defined with Nanodrop ND-2000C (Thermo Scientific, Waltham, MA, USA).

For microarray hybridization one µg of the biotinylated cRNA sample was mixed with the Hyb E1 hybridizatioin buffer containing 37.5% (w/w) formamide and then hybridized to Sentrix Bead Chip Human HT12_v4 (Illumina, Inc., San Diego, CA) at 58 °C overnight (18 hrs). The array represents over 47,000 bead types, each with a unique sequence derived from human genes in the National Centre for Biotechnology Information Reference Sequence and UniGene database. Array chips were washed with manufacturer’s E1BC solution, stained with 1 ug/mL Cy3-streptavidine (Amersham Biosciences) and eventually scanned with Illumina BeadArray Reader.

1. **Supplementary file**

**The xls files contain the complete list of DE genes in the three subtypes:**

- **6-DE genes in CC cond BCAF vs cond NHDF (FC1.5 Pval1E-04).xlsx (class comparison between treatment with CM by CAF vs NAF)**
- **6-DE genes in CC cond BCAF vs Ctrl (FC1.5 Pval1E-04).xlsx (class comparison between treatment with CM by CAF vs CTRL medium)**
- **6-DE genes in CC cond NHDF vs Ctrl (FC1.5 Pval1E-04).xlsx (class comparison between treatment with CM by NAF vs CTRL medium)**

1. **Supplementary Figures**

The file contains Figure S1 (Kaplan-Meier curve) and Figure S2 (heat-map)

1. **Legends to Supplementary Figures**

**Figure S1. *Kaplan-Meier curves comparing distant metastasis-free survival (DMFS) according to µENV status defined using non-subtype-specific µENV signatures***. The number of patients in each group at each time is reported. For each comparison hazard ratio (HR), 95% CI and *P-*value are reported.

**Figure S2. Heat map summarizing IL-8 and IL-6 levels in conditioned media (CM) from monotypic and heterotypic cell cultures.**

Cytokine concentrations are represented by independent color codes. Light yellow (low levels) to dark orange (high level) is used IL-8 levels; light yellow (low level) to dark green (high level) is used for IL-6. Absolute concentrations, obtained by ELISA and expressed in pg/ml are also reported.

1. **Supplementary Tables**

The file contains:

Table S1 (Overlap between the µENV signatures and published microenvironment-related signatures)

Table S2 (Patient numbers and number of unfavorable events in the publicly available gene expression collections.

Table S (Results of test based on Schoenfeld residuals for checking proportional hazards in multivariable Cox analysis for lymph-node negative untreated patients with ESR1+/ERBB2- tumors
